# Assessing Organoleptic Properties Simulation of a Placebo of Raw Food Material to a Chinese Medicine Oral Liquid FYTF‐919

**DOI:** 10.1155/bmri/9000068

**Published:** 2026-08-30

**Authors:** Wanzhen Cui, Daojin Xue, Wenwei Ouyang, Yang Zhao, Meiling Xuan, Li Zhou, Guo Ke, Yimeng Gao, Linghui Kong, Yajie Chen, Juan Huang, Haiding Huang, Gang Lu, Liling Zeng, Tao Huang, Lili Song, Jianwen Guo, Zehuai Wen

**Affiliations:** ^1^ Second School of Clinical Medicine, Guangzhou University of Chinese Medicine, Guangzhou, China, gzucm.edu.cn; ^2^ Department of Neurosurgery, Guangdong Provincial Hospital of Chinese Medicine, Guangzhou, China, gdhtcm.com; ^3^ Key Unit of Methodology in Clinical Research, Guangdong Provincial Hospital of Chinese Medicine, Guangzhou, China, gdhtcm.com; ^4^ Department of Global Public Health, Karolinska Institute, Stockholm, Sweden, ki.se; ^5^ George Institute for Global Health, Beijing, China; ^6^ Guangdong Huixiangyuan Biotechnology Co. LTD, Guangzhou, China; ^7^ Key Laboratory of Quality Evaluation of Chinese Medicine of the Guangdong Provincial Medical Products Administration, Guangdong Provincial Hospital of Chinese Medicine, Guangzhou, China, gdhtcm.com; ^8^ Public laboratory, Guangdong Provincial Hospital of Chinese Medicine, Guangzhou, China, gdhtcm.com; ^9^ CUHK-SDU Joint Laboratory on Reproductive Genetics, School of Biomedical Sciences, The Chinese University of Hong Kong, Hong Kong, China, cuhk.edu.hk; ^10^ Department of Neurology, Guangdong Provincial Hospital of Chinese Medicine, Guangzhou, China, gdhtcm.com; ^11^ State Key Laboratory of Dampness Syndrome of Chinese Medicine, Guangdong Provincial Hospital of Chinese Medicine, Guangzhou, Guangdong, China, gdhtcm.com

**Keywords:** blinding and physical consistency, clinical trial, objective evaluation, sensory evaluation, subjective evaluation

## Abstract

**Background:**

Research on blinding and physical consistency between placebo and Chinese medicine is in early stages. We complement novel methodologies for assessing blinding and physical consistency meanwhile investigate different roles of the two evaluations, taking Chinese botanical drug FYTF‐919 with matched placebo as an example.

**Methods:**

Subjective evaluation through a clinical trial and objective evaluation by intelligent sensory analysis technology were used in this study. Eligible individuals were recruited and randomly provided FYTF‐919 or placebo. Participants were instructed to determine nature of the substance (FYTF‐919 or placebo) and compare odor and taste of the given substance with true FYTF‐919. Objective evaluation on odor and taste properties was conducted by E‐nose and E‐tongue.

**Results:**

Forty‐seven participants completed this trial. More than half participants identified placebo as FYTF‐919; comparable proportions of participants in each group identified testing drug as FYTF‐919. There’s no significant difference in taste score between two groups. Higher odor score for FYTF‐919 was associated with medical experience. Consistent with the clinical trial findings, intelligent machines‐based sensory evaluation revealed that the two drugs could be confused in taste, whereas their odors were distinguishable.

**Conclusions:**

The placebo was effectively blinded to the traditional Chinese medicine in this study. However, the similarity between the drug and the placebo has not been fully confirmed, as their odor might be detected by the E‐nose and medical staff. Therefore, medicine dispensing by dedicated personnel and preventing medical staff from having access to the investigational drugs are necessary for successful blinding throughout the clinical trial.

**Trial Registration:** ClinicalTrials.gov identifier: NCT05483595

## 1. Introduction

In modern medicine, double‐blinded randomized controlled trials (RCTs) are believed to provide the highest level of evidence [1, 2]. A critical methodological feature of double‐blinded RCTs is the implementation of blinding [3–6], which has been incorporated into the Consolidated Standards of Reporting Trials (CONSORT) statement [7]. Successful blinding can control some biases, including expectation bias, response bias, and observation bias, in participants and investigators [8–10]. Over the past several decades, RCTs of traditional Chinese medicine (TCM) have become increasingly popular. However, most published studies have not evaluated blinding.

Placebo plays an important role in blinded RCTs. A suitable placebo facilitates blinding by controlling all potential influences, including spontaneous changes, participant or investigator expectations, and the Hawthorne effect [11]. Poor placebo features can affect the blinding success and lead to unreliable study results [12]. Therefore, identical physical characteristics between placebo and investigational drug are an important guarantee for the implementation of blinding. However, no more than 15% of the TCM placebo‐controlled trials registered in the World Health Organization′s (WHO) International Clinical Trials Registry (ICTRP) reported the identical physical appearance of the placebo and TCM; and none reported the testing methods for determining whether the placebo was physically identical to the TCM [12].

According to the specification for evaluation of simulation effect of Chinese patent medicine placebo in 2022, subjective evaluation methods including independent evaluation and comparative evaluation should be used for similarity evaluation of placebo [13]. The independent evaluation, where each person is given only experimental drug or placebo in a blind state, gives an overall assessment of organoleptic properties, whereas the comparative evaluation is to evaluate single sensory properties between placebo and investigational drug by simulating clinical scenarios in which researchers or patients are exposed to the placebo and the testing drug at the same time [14]. Zhang et al. proposed a combination of subjective scoring and objective evaluation for the physical consistency assessment in placebo‐controlled TCM intervention trials [12]. Different from participants′ evaluation, intelligent sensory evaluation including electronic nose (E‐nose) and electronic tongue (E‐tongue) which are composed of multidimensional parameters can objectively reflect the sensory properties of TCM and placebo, reducing subjective bias. There is no case of combining participants′ evaluation with intelligent sensory evaluation to evaluate the simulation effect of TCM placebo.

This study was designed to evaluate the simulation effect of a Chinese patent medicine placebo in the trial of Chinese Herbal medicine in patients with Acute INtracerebral hemorrhage (CHAIN) (ClinicalTrials.gov Identifier: NCT05066620). Independent evaluation was used to determine whether participants could identify the Zhongfeng Xingnao oral liquid (FYTF‐919) or the matched placebo used in the CHAIN trial. A comparative evaluation and intelligent sensory analysis technology were used to assess the physical properties of FYTF‐919 and matched placebo.

## 2. Materials and Methods

### 2.1. Study Design

**Figure 1:**
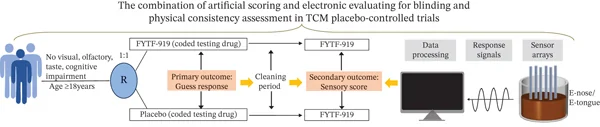
Flow chart of this study. R, randomization.

This study is aimed at evaluating the blinding and physical consistency between the placebo and FYTF‐919. The blinding evaluation refers to an independent assessment, whereas the physical consistency assessment involves a comparative evaluation of the odor and taste properties between FYTF‐919 and placebo. Because the matched placebo of Chinese botanical drug oral liquid can almost not be completely consistent with the investigational drug in the organoleptic evaluation, as long as this trial is impossible to distinguish between placebo and FYTF‐919, the blinding can be achieved. That is, the study design considered logically that the proportion of FYTF‐919 and placebo being correctly identified is not significantly greater than the chance of random guess (50%), which means that FYTF‐919 and placebo cannot be accurately distinguished in the trial, and the blinding process in the trial can be achieved [14–16]. Numerical rating scale (NRS) score ranged from 0 to 10 reflects participants′ subjective assessment for the odor and taste consistency between FYTF‐919 and placebo, which is consistent with the score evaluation method proposed by China Association of Chinese Medicine (CACM) [13]. Potential participants were recruited to be screened, and eligible individuals were randomly assigned at a ratio of 1:1 to receive either FYTF‐919 or a placebo. First, each participant was given a coded testing drug to determine whether it was FYTF‐919 or placebo. Subsequently, they had a cleaning period of several minutes to drink or gargle with purified water to remove any residue of the drug they had just assessed. They were then provided FYTF‐919 to evaluate its consistency with the former drug regarding odor and taste (Figure 1).

In addition to the clinical trial, the auxiliary E‐nose and E‐tongue were used to detect placebo and FYTF‐919 to obtain objective data on odor‐ and taste‐related parameters.

This study was approved by the local ethics committee. All participants provided written informed consent before participating in the study. The data for this study will also be available from the principal investigator upon reasonable request.

### 2.2. Participants

Potential participants, including medical workers, patients, and their families, were recruited through notice boards and inpatient visits from Guangdong Provincial Hospital of Chinese Medicine (GPHCM) in Guangdong, China. Follow‐up was conducted at the outpatient or inpatient department to collect the questionnaire data. Participants aged ≥ 18 years were eligible for enrollment if two further criteria were met: (1) no visual, olfactory, taste, or cognitive impairment, and (2) each provided written informed consent. Exclusion criteria included individuals who were evaluation personnel involved in the clinical trial for FYTF‐919; participants with a history of severe heart, liver, kidney, digestive tract, nervous system, respiratory system, mental disorders, or metabolic disorders; participants with a history of food or drug allergies, or other allergic diseases (asthma, urticaria, eczematous dermatitis, etc.), or who was known to be allergic to FYTF‐919; or for whom Chinese botanical drugs were contra‐indicated; women who were pregnant or lactating; and those not fit to participate in this study as judged by the clinician.

### 2.3. Randomization and Blinding

The testing drugs were packaged by an assistant who was not involved in any other aspect of the study, according to a random allocation table created using SAS 9.2 software (SAS Institute Inc., Cary, United States). All drugs were packaged identically in shape, specification, appearance, packaging, labeling, and other controllable aspects, except for the randomly blinded bottle number code. All the participants, investigators, and statisticians were blinded to the grouping and allocation. Participant allocation was executed through a verified, web‐based interactive response system for the Chinese medicine trial. When a participant was confirmed eligible, the personal information was entered into the system to complete the randomization and receive a blinded bottle number. Ultimately, all participants were randomly allocated at a ratio of 1:1 to a group that received FYTF‐919 or placebo.

### 2.4. Chinese Botanical Drug Oral Liquid and Placebo

Chinese botanical drugs FYTF‐919 is developed by Hospital of Chengdu University of Traditional Chinese Medicine, Sichuan, China, which has not only obtained the approval number of Z20070623 by medical institutions in Sichuan Province, but also obtained its own Chinese patent number of ZL01823262.0 and international PCT patent number of A61K35/78. FYTF‐919, composed of *Panax ginseng* C.A.Mey (Araliaceae; Ginseng radix et rhizoma), *Panax notoginseng* (Burkill) F.H.Chen (Araliaceae; Notoginseng radix et rhizoma), *Conioselinum anthriscoides* “Chuanxiong” (Apiaceae; chuanxiong rhizoma), and *Rheum officinale* Baill (Polygonaceae; Rhei radix et rhizoma) at a ratio of 2:2:2:1, is prepared by decocting and boiling in water, with 70 g of the above ingredients per 100 mL. According to the manufacturer′s instructions, the Chinese botanical drug preparations are used to treat stroke at a dose of 25 mL four times a day or 30 mL three times a day. The FYTF‐919 used in this trial was produced by the Affiliated Hospital of Chengdu University of Traditional Chinese Medicine (Batch Number 20211210).

In our previous work, we have conducted quality control tests on FYTF‐919. Firstly, we conducted chromatographic fingerprint compared the information of molecular formula, molecular weight, peak time and secondary fragment ions with reference substances, databases and related literatures. Chromatographic fingerprint similarity analysis indicated highly consistent peaks and satisfactory batch‐to‐batch stability of FYTF‐919. Then ultrahigh performance liquid chromatography coupled with a Q exactive hybrid quadrupole‐orbitrap mass spectrometry system (UHPLC‐Q‐Exactive‐Orbitrap MS) was employed to perform the qualitative and quantitative analysis of FYTF‐919. As a result, a total of 30 components in FYTF‐919 have been identified or tentatively identified eventually, and the representative components include ginsenoside Re, ginsenoside Rg1, ginsenoside Rb1, notoginsenoside R1, emodin‐8‐O‐*β*‐D‐glucoside, rhein, and ferulic acid, and so forth [17]. In addition, methodological validation, including precision validation and linear regression analysis, demonstrated satisfactory precision and reliability of the established analytical method, and developed seven excellent linear relationships as quantitative analysis criteria. Detailed methods and results of quality control for FYTF‐919 were provided in the Supporting Information (File S1).

The placebo was manufactured by the Guangdong Huixiangyuan Biotechnology Co. LTD (Batch Number 22.06.02‐03). The following features of appearance and the internal laws of flavor level structure, color and gloss, texture, basic flavor, main flavor, and volatile aroma of FYTF‐919 were imitated by the placebo, which was composed of food‐grade and medicinal‐valueless materials, such as soy protein powder, vital wheat gluten, and brown sugar syrup (Table 1). Invention patents based on the placebos were published by the State Intellectual Property Office of China (Patent Number ZL 202211384394.6).

**Table 1:** Features of FYTF‐919 and ingredients of placebo.

Items	FYTF‐919	Placebo
Color and gloss	Dark black	Brown sugar syrup, food essence
Texture	Slightly viscous	Maltodextrin
	Homogeneous	Vital wheat gluten
Basic flavor	Sweet	Brown sugar syrup, stevioside
	Bitter	Soy protein powder
	Sour	DL‐malic acid
Main flavor	Neem wood	Food essence
Volatile aroma	Aroma of rotting wood	Food essence
	Earthy smell	Soy protein powder, maltodextrin

**Figure 2:**
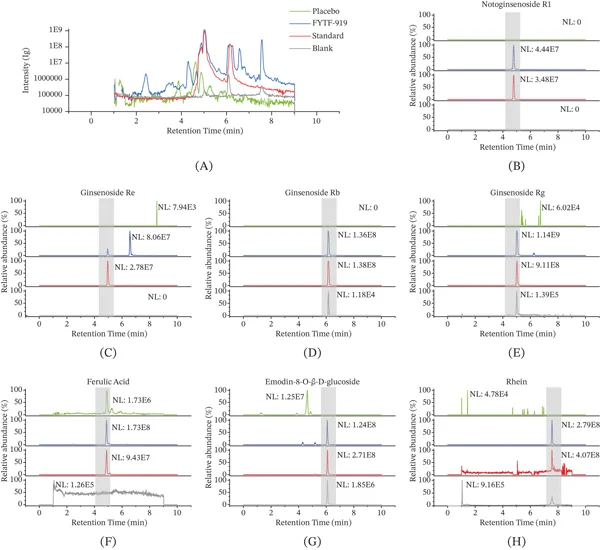
The exacted ion chromatograms (EICs) of FYTF‐919‐specific active components in different samples. (A) Overlay of EICs for placebo (green), FYTF‐919 (blue), standard (red), and blank (black). (B–H) EICs for the target analytes in different groups. NL indicates the normalization level of relative abundance. Shaded boxes highlight the characteristic peaks of each analyte. The specific retention times (*t*_*R*_) and monitored *m/z* ranges used for identification were as follows: (B) Notoginsenoside R_1_(*t*_*R*_4.78 min, *m/z* 931.5173–931.5359); (C) ginsenoside Re (*t*_*R*_4.97 min, *m/z* 945.5318–945.5508); (D) ginsenoside Rb_1_ (*t*_*R*_ 6.15 min, *m/z* 1107.5793–1107.6015); (E) ginsenoside Rg_1_ (*t*_*R*_ 5.03 min, *m/z* 845.4823–845.4993); (F) ferulic acid (*t*_*R*_ 4.88 min, *m/z* 193.0479–193.0517); (G) emodin‐8‐ O ‐ *β* ‐D‐glucoside (*t*_*R*_ 6.10 min, *m/z* 431.0948–431.1034); and (H) rhein (*t*_*R*_ 7.57 min, *m/z* 283.0224–283.0280).

To verify the absence of FYTF‐919‐specific active components in the placebo, qualitative detection was conducted using the identical UHPLC‐Q‐Exactive‐Orbitrap MS conditions across four groups: blank, standard, FYTF‐919, and placebo. As shown in Figure 2, the exacted ion chromatograms (EICs) for target analytes revealed high resolution and intensity in the standard and FYTF‐919 groups. In contrast, the blank group showed minimal background interference. Based on the dual identification criteria of retention time and base peak m/z, the EICs of FYTF‐919 confirmed the presence of all target components, matching the standards. Conversely, no target analytes were detected in the placebo group. In conclusion, the placebo sample is completely free of FYTF‐919‐specific active components.

This study used a determined and consistent 30 mL dose of the drug for each test. At the end of the trial, all the investigational drugs were retrieved and destroyed.

### 2.5. Outcome Measurements

The primary outcome was the percentage of the FYTF‐919 guess response. Secondary outcomes included the artificial sensory scores of the test drug. A subanalysis was conducted to investigate the sensory scores of medical workers and others as well as the proportion of FYTF‐919 guess responses in medical workers.

We also evaluated the two sensory dimensions using an E‐nose and E‐tongue. The sensor arrays of the E‐nose and E‐tongue consisted of 14 and 6 sensors, respectively (Tables S1 and S2). Those in the E‐nose category generated response signals to different volatile compounds, and sensors equipped in the E‐tongue provided global taste profiles and specific taste perception [18, 19].

### 2.6. Data Collection and Management

Each participant was provided with a data collection form to record demographic information, medical history, global judgment, and manual physical consistency scores. The odor and taste scores of the placebo and FYTF‐919 were comprehensively evaluated, serving as raw data for the concordance or similarity comparison between FYTF‐919 and placebo. To clarify this, concordance or similarity was evaluated using the NRS score, which ranged from 0 to 10, with 0 being completely inconsistent and 10 being completely consistent. The completion of the scale was used to monitor participant compliance.

Objective data on odor‐ and taste‐related parameters was obtained by E‐nose and E‐tongue. The E‐nose (Isenso Intelligent, Shanghai, China) was equipped with an array of metal oxide semiconductor sensors, which consisted of 14 sensors and could generate different response signals to different volatile compounds. Before the detection, a 10 mL sample of FYTF‐919 or placebo was accurately transferred into a 40 mL headspace vial. Then the vials were equilibrated at 50°C for 30 min. After that, run the detection program, which lasts for 60 s. Clean the headspace vial after each detection for 60 s to ensure thorough cleaning. Each sample was tested in eight replicates, generating eight sets of 14‐dimensional data across the 14 sensors. The E‐tongue was purchased from Shanghai Bosin Industrial Development Co. Ltd and was equipped with six metallic sensors (Platinum, Gold, Palladium, Tungsten, Titanium, and Silver). Each sensor could generate three‐dimensional response values. A 20 mL sample of FYTF‐919 or placebo was accurately transferred into a 50 mL beaker for test and the detection cycle lasted for 120 s. The sensors signal amplification was set to 100 times. Each sample was tested in eight replicates, generating eight sets of 18‐dimensional data across the six sensors.

### 2.7. Statistical Analysis

We assume that the correct response rate of FYTF‐919 is 50% and not more than 70%, whereas a previously reported study [15] suggested that the correct response rate of Chinese botanical drug could reach 70% and 50% of placebo (that is, the chance of guessing). Therefore, the estimated sample size was 48 cases, which was determined to provide 80% power to achieve statistical significance at a 5% two‐sided level with a 20% margin of noninferiority. We did not consider participant loss to follow‐up because participants enrolled the trial for evaluation immediately after informed consent. Participants underwent only one evaluation visit and were not expected to be lost to follow‐up; thus, missing data processing was not considered. If extreme values occurred, baseline checks were required.

The data were supervised for quality control by the GPHCM methodology team. Descriptive statistics, including means with standard deviation (SD) for continuous variables and counts and percentages for categorical variables were calculated. The primary outcome was analyzed using the two‐proportion hypothesis to investigate the differences between the binomial proportions. A subgroup analysis of the odor scores assessed by medical workers and patients/family members was also performed. For the secondary outcomes, a nonparametric test was used for continuous variables. Univariate regression analysis was used for the correlation analysis. Proportional data were analyzed using the same strategy as for the primary outcome. Two‐tailed *p* < 0.05 were considered statistically significant.

In addition, intelligent sensory data of FYTF‐919 and placebo were obtained from the E‐nose and E‐tongue sensors for transforming and reducing dimensions, followed by linear classification, that is, principal component analysis (PCA). PCA was used to visualize the data structures of the different intelligent sensory technologies.

## 3. Results

### 3.1. Participant Characteristics Comparison

Forty‐eight eligible participants from the GPHCM were randomly assigned to the FYTF‐919 and placebo groups at an equal ratio. Except for one participant in the FYTF‐919 group who withdrew informed consent before the trial, 47 completed the study. There were no significant differences between the two groups regarding age, sex, participant category, or previous use of TCM (Table 2).

**Table 2:** Participant characteristics.

Characteristics		Group	
		FYTF‐919 (*n* = 23)	Placebo (*n* = 24)
Age^b^, mean ± SD, y		39.70 ± 14.54	45.63 ± 22.17
Sex^a^, *n* (%)	Male	11 (47.83)	9 (37.5)
	Female	12 (52.17)	15 (62.5)
Category of subjects^a^, *n* (%)	Medical workers	12 (52.2)	12 (50.0)
	Patient/families	11 (47.8)	12 (50.0)
History use of TCM^a^, *n* (%)	No	16 (69.6)	19 (79.2)
	Yes	7 (30.4)	5 (20.8)

*Note:* Data are mean ± SD or *n* (%).

Abbreviations: SD, standard deviation; TCM, traditional Chinese medicine.

^a^By *χ* ^2^ test.

^b^By *t* test.

### 3.2. Primary Outcome

**Figure 3:**
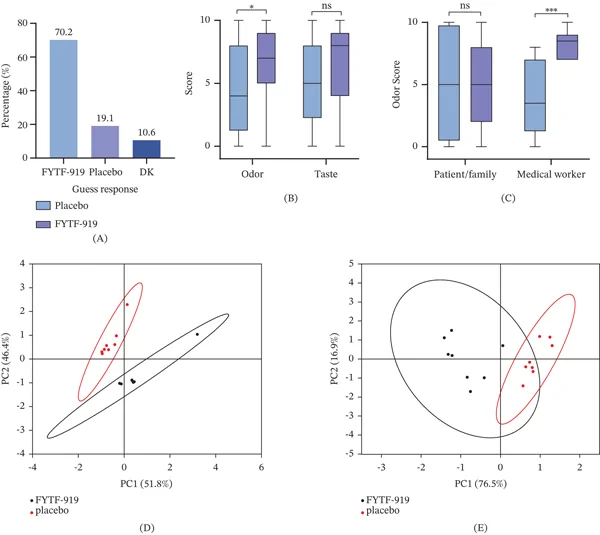
(A) Proportion of all participants′ responses to the testing drug. (B) NRS scores of odor and taste for all participants. (C) NRS scores of odor for patients/families and medical workers. (D) PCA of sensory attributes by E‐nose. (E) PCA of sensory attributes by E‐tongue. ∗*p* < 0.05, ∗∗∗*p* < 0.001, by Mann–Whitney test; NRS, numerical rating scale; PCA, principal component analysis.

Each participant was required to determine whether the given drug was “FYTF‐919” or “Placebo” and “Do not know” (DK) responses. We present the proportional response for all the participants, more of whom (70.2%) tended to perceive the test drug as FYTF‐919 than the placebo (Figure 3A). There is no significant difference in the proportion of participants guessing FYTF‐919 between the two groups (65.2% vs. 75.0%, respectively; *p* = 0.534, Table 3). Although the wide confidence interval (95% CI [−0.36, 0.16]) reflects the limited sample size and precludes a definitive statistical claim of equivalence, the observed distribution indicated that participants could not reliably distinguish between FYTF‐919 and placebo. Thus, the blinding appears to have been maintained effectively within the limits of this study′s power.

**Table 3:** Participants′ response in terms of frequency.

Group	Guess response, *n*(%)		
	FYTF‐919	Placebo	DK
FYTF‐919	15 (65.2)	6 (26.1)	2 (8.7)
Placebo	18 (75.0)	3 (12.5)	3 (12.5)
*Z* = −0.74, *p* = 0.534, 95% CI [−0.36, 0.16]			

*Note:* Data are shown as *n* (%), by two‐proportion hypothesis.

Abbreviation: DK, do not know.

### 3.3. Secondary Outcomes

In terms of sensory assessment, as expected, the FYTF‐919 group exhibited higher median NRS scores for odor and taste. Nonparametric tests revealed no significant differences between the two drugs for the taste scores (*p* = 0.144), whereas significant differences were found for odor (*p* = 0.025, Figure 3B).

The result of PCA showed that the first two principal components explained 98.2% of the variance in the E‐nose data and 93.4% of that in the E‐tongue data, respectively. The PCA of the E‐tongue data indicated a partial overlap between the FYTF‐919 and placebo groups, whereas the PCA of the E‐nose data showed a clear clustering phenomenon between the two groups (Figure 3D,E). The results of the sensory evaluation by participants or intelligent machines indicated that there was good confusion in terms of taste, but not odor, regarding the two drugs.

Given that discrimination between TCM and placebo based on sensory evaluation may be influenced by the participants′ characteristics, such as their TCM experience, we explored the effects of age, sex, history of TCM use, and medical experience on their odor and taste judgment for testing drugs. Notably, although medical workers rated the odor of the placebo as similar to that of patients or family members (*p* = 0.431, Table S3), they tended to give a higher odor score to FYTF‐919 than the latter (*B* = 3.333, *p* = 0.004, Table 4). There was no significant difference in taste ratings for either drug between medical workers and the patients or family members (*p* = 0.245, Tables S4 and *p* = 0.211, Tables S5). Furthermore, factors, such as age, sex, and previous use of TCM, had minimal influence on the sensory scores for both drugs (*p* > 0.05, Table 4 and Tables S3, S4, S5).

**Table 4:** Univariate regression analysis of factors influencing FYTF‐919 odor score.

Variables	B	95% CI	*p*
Age	−0.027	−0.119, 0.064	0.539
Male	0.152	−2.475, 2.778	0.906
Medical worker	3.333	1.185, 5.482	0.004 ^∗^
History use of TCM	1.063	−1.749, 3.874	0.441

Abbreviation: TCM, traditional Chinese medicine.

^∗^ *p* < 0.05.

As medical workers rated higher odor scores for FYTF‐919 than did patients/family members, we analyzed the odor scores of medical workers versus patients or family members separately. The odor scores of patients or family members were similar between the placebo and FYTF‐919 groups. However, the scores rated by medical staff differed significantly between the two groups (Figure 3C). We then analyzed the medical workers′ responses to the test drugs and found that a consistent proportion of medical workers in the two groups identified the test drug as FYTF‐919 (Table 5). It indicated that for odor, participants or family members failed to reliably distinguish FYTF‐919 from the placebo, whereas medical workers with extensive TCM exposure might identify the two drugs. Although the placebo did not closely mimic the odor of FYTF‐919, the high percentage of medical workers guessing FYTF‐919 suggested that their prior knowledge and experience did not compromise the integrity of the blinding as they were unable to leverage such factors to correctly identify their group allocation.

**Table 5:** Medical workers′ response in terms of frequency.

Group	Guess response, *n*(%)		
	FYTF‐919	Placebo	DK
FYTF‐919	8 (66.7)	3 (25)	1 (8.3)
Placebo	8 (66.7)	2 (16.7)	2 (16.7)
*Z* = 0, *p* = 1.00, 95% CI [−0.38, 0.38]			

*Note:* Data are shown as *n* (%); by two‐proportion hypothesis.

Abbreviation: DK, do not know.

## 4. Discussion

Specialized preparation techniques contribute to the unique appearance, smell, and taste of TCM [20], making it difficult to manufacture a placebo that can perfectly mimic these characteristics. This may explain why only a few studies have evaluated the physical consistency between the placebo and formulated products [21]. It is imperative to assess the success of blinding before evaluating clinical trial results. This can serve as a normative measure for assessing the quality of TCM‐matched placebo and simultaneously assure subsequent trials. Researchers usually assess blinding by comparing the proportion of correct or incorrect guess responses between the medication groups [15]. Successful blinding is derived from a similar or higher proportion of TCM guess responses in the placebo group than in the TCM group [15]. Therefore, we conducted this trial to evaluate the blinding of the placebo. In this trial, there was no statistically significant difference in the proportion of “FYTF‐919 guess responses” between the FYTF‐919 and placebo groups. Although the wide confidence interval reflects the limited sample size and precludes a definitive claim of statistical equivalence, the observed distribution indicated that the participants in both groups could not clearly distinguish between FYTF‐919 and the matched placebo, that is, within the constraints of the study’s power, the blinding appears to have been effectively maintained. The same results were observed in the medical worker subgroup, suggesting that prior medical knowledge did not compromise the integrity of the blinding.

Physical consistency assessment has been suggested in the CONSORT extension for Chinese herbal medicine formulas 2017 [7] and the WHO trial registration dataset for TCM extension 2020 [22]. According to the studies registered in the WHO ICTRP, Zhang et al. proposed a combination of subjective scoring and objective evaluation for the physical consistency assessment in placebo‐controlled TCM trials [12]. In our study, both subjective scoring and intelligent electronic evaluation were conducted to assess the physical consistency between FYTF‐919 and a matched placebo. For comparison, we included two sensory dimensions: odor and taste. The two groups′ NRS scores for odor and taste were also performed. The results showed that the placebo was similar to FYTF‐919 in taste but did not completely mimic FYTF‐919 in odor. The multidimensional data for the E‐nose and E‐tongue showed the same results. Correlation analysis revealed that the high odor scores of FYTF‐919 were associated with medical workers with extensive exposure to TCM. Further analysis revealed that the placebo was confused with FYTF‐919 regarding odor for the patients or family members but not for medical workers.

The CACM in 2022 proposes some manual evaluation methods for the physical consistency assessment of placebo with Chinese patent medicine [13]. Xiao et al. proposed that the higher the degree of randomization, the less subjective factors would influence the evaluation results, thus making them more reliable [14]. Our study proposed a new scenario: Participants were randomly provided either TCM or placebo in a blind state to determine the nature of the substance (whether it was TCM or placebo); then they were provided TCM to evaluate its consistency in terms of odor and taste with the former substance. The assessment of the placebo simulation effect in this study was conducted based on the principles of randomization and blinding. The intelligent evaluation of organoleptic properties between TCM and placebo yielded consistent results with participants′ evaluation, indicating the reliability of the research findings. These methodologies can be applied to assess the placebo simulation effect of TCM in other similar studies.

In our study, the RCT‐based blinding test was essentially a pretrial on the blinding effect of the intervention clinical trial; thus, the results of the blinding test were considered the primary reference. Physical consistency tests allowed the participants or instruments to repeatedly compare the placebo with TCM to complete multidimensional sensory assessments, which were used to identify the factors that may cause unblinding and further guide implementation of blinding in formal clinical trials. The results of simulation effect demonstrated that the placebo used in the CHAIN trial for blinding was feasible. It is necessary to appoint special personnel to dispense drugs to patients in the CHAIN trial, which prevents medical workers from having access to intervention drugs and plays an extremely important role in the quality control of RCT with TCM interventions.

## 5. Conclusions

This is a preliminary study that proposes novel methods to combine blinding evaluation with physical consistency evaluation in the assessment of the simulation effect of TCM placebo, and the results demonstrated that the placebo used in the CHAIN trial was feasible for blinding. However, physical consistency between the drug and the placebo has not been fully confirmed, as their odors might be detected by the E‐nose and medical staff. Therefore, it is necessary to appoint special personnel to dispense drugs to patients in the CHAIN trial, which prevents medical workers from having access to intervention drugs and plays an extremely important role in the quality control of RCT with TCM interventions.

The different roles of diverse detection modalities are summarized. Integrating subjective and objective evaluations can reduce subjective bias and enhance the credibility and reliability of evaluation. Blinding evaluation predicts the success rate of blinding. Physical consistency evaluation identifies factors that could compromise blinding and allows for pre‐emptive measures, thus enhancing blinding effectiveness. The methods for assessing blinding and physical consistency in this study can be applied to other TCM versus placebo studies and the combination of the two assessments is indispensable for a better blinding protocol.

**Nomenclature**
CACM: China Association of Chinese Medicine
CHAIN: Chinese Herbal medicine in patients with Acute INtracerebral hemorrhage trial
CONSORT: Consolidated Standards of Reporting Trials
E‐nose: electronic nose
E‐tongue: electronic tongue
EICs: exacted ion chromatograms
FYTF‐919: Zhongfeng Xingnao oral liquid
GPHCM: Guangdong Provincial Hospital of Chinese Medicine
ICTRP: International Clinical Trials Research Platform
NRS: numerical rating scale
PCA: principal component analysis
RCT: randomized controlled trial
SD: standard deviation
TCM: traditional Chinese medicine
UHPLC‐Q‐Exactive‐Orbitrap MS: ultrahigh performance liquid chromatography coupled with a Q exactive hybrid quadrupole‐orbitrap mass spectrometry
WHO: World Health Organization

## Author Contributions

**Wanzhen Cui:** data curation, formal analysis, writing – original draft. **Daojin Xue:** data curation, formal analysis, writing – original draft. **Wenwei Ouyang:** formal analysis, writing – original draft. **Yang Zhao:** supervision. **Meiling Xuan:** methodology, validation, supervision, data curation. **Li Zhou:** data curation. **Guo Ke:** investigation. **Yimeng Gao:** investigation. **Linghui Kong:** resources, supervision. **Yajie Chen:** supervision. **Juan Huang:** investigation, visualization. **Haiding Huang:** investigation, visualization, funding acquisition. **Gang Lu:** project administration, supervision. **Liling Zeng:** project administration, supervision, funding acquisition. **Tao Huang:** conceptualization, methodology, funding acquisition. **Lili Song:** conceptualization, methodology. **Jianwen Guo:** project administration, conceptualization, methodology, funding acquisition. **Zehuai Wen:** conceptualization, methodology, writing – review and editing. **Wanzhen Cui**, **Daojin Xue**, and **Wenwei Ouyang** have contributed to the work equally and should be regarded as co‐first authors.

## Funding

This study was supported by the Guangdong Provincial Department of Science and Technology (10.13039/501100007162, 2020B1111100009); National Natural Science Foundation of China (10.13039/501100001809, 81974559); Guangzhou Municipal Science and Technology Bureau (10.13039/501100020084, 202102010287, 202201011302); Guangzhou Traditional Chinese Medicine and Integrative Medicine research project (20222A011010); The 17th Top Talent Guangdong Provincial Hospital of Chinese Medicine (BJ2022YL06); and The 13th Zhaoyang Talent of Guangdong Hospital of Chinese Medicine (ZY2022YL28).

## Disclosure

All authors have read and approved the final manuscript.

## Ethics Statement

This trial was approved by ethics committee of Guangdong Provincial Hospital of Chinese Medicine (Approval Number: BE‐2022‐187‐01).

## Consent

Consent is not applicable and no identifying images or other personal or clinical details of participants are presented here or will be presented in reports of the trial results.

## Conflicts of Interest

The authors declare no conflicts of interest.

## Supporting Information

Additional supporting information can be found online in the Supporting Information section.

## Supporting information


**Supporting Information 1** Table S1: Sensors and the corresponding volatile components of E‐nose. Table S2: Sensors and corresponding taste profiles of E‐tongue. Table S3: Univariate regression analysis of factors influencing placebo odor score. Table S4: Univariate regression analysis of factors influencing placebo taste score. Table S5: Univariate regression analysis of factors influencing FYTF‐919 taste score.

**Supporting Information 2** File S1: Quality control of FYTF‐919 based on UHPLC‐Q‐Exactive‐Orbitrap MS.

## Data Availability

The datasets used and/or analyzed during the current study are available from the corresponding authors on reasonable request.
