# Supplementary material for: Assessing Organoleptic Properties Simulation of a Placebo of Raw Food Material to a Chinese Medicine Oral Liquid FYTF‐919
Source: Biomed Res Int. 2026 Aug 30;2026:9000068. doi: 10.1155/bmri/9000068 (PMC13527160; doi:10.1155/bmri/9000068)
Supplement: Supplementary file 1 [file BMRI-2026-9000068-s001.docx]

**File S1. Quality control of FYTF-919 based on UHPLC-Q-Exactive-Orbitrap MS**

**1.Materials**

**1.1 Instruments**

Thermo UltiMate 3000 UHPLC instrument (Waltham, MA, USA). Q-Exactive hybrid quadrupole-orbitrap mass spectrometer (Thermo Scientific, San Jose, USA), LSE Vortex Mixer (Corning Incorporated, USA), LK/CSJ-20 Ultrasonic Cleaner (Chengdu Laoken Medical Technology Co., LTD.), 5430R High-speed refrigerated Centrifuge (Eppendorf, Germany), Pure water machine (Guangzhou Yuwei Biological Technology Co., LTD.), BT-125D Electronic Balance (Sartorius Scientific Instruments (Beijing) Co., LTD.)

**1.2 Reagents and Materials**

There were 3 batches of FYTF-919 samples (batch numbers 202109.6、20211210、20220419). All standard substances(>98% purity), namely neochlorogenic acid, chlorogenic acid, emodin-8-O-β-D-glucoside, physcion-8-O-β-D-monoglucoside, glycitin, cryptochlorogenic acid, isochlorogenic acid A, isochlorogenic acid B, isochlorogenic acid C, ginsenoside Rg2, 20(R)-ginsenoside Rg2, ginsenoside Rc, ginsenoside Rg3, 20(R)-ginsenoside Rg3, ginsenoside Rb2, ginsenoside Rf, ginsenoside Rh1, 20(R)-ginseno side Rh1, 20(R)-notoginsenoside R2, 20(S)-notoginsenoside R2, and chrysophanol 1-glucoside were purchased from Shanghai yuanye Bio-Technology Co. Ltd. (Shanghai, China).

The following compounds, namely ferulic acid (99.4% purity), notoginsenoside R1 (94.0% purity), ginsenosides Re (96.0% purity), ginsenosides Rg1 (98.5% purity), ginsenosides Rb1 (95.1% purity), and ginsenosides Rd (97.3% purity), aloe-emodin (98.3% purity), rhein (99.3% purity), emodin (96.0% purity), gallic acid (91.5% purity), and chrysophanol (99.4% purity) were purchased from China National Institutes for Food and Drug Control.

HPLC-grade formic acid, methanol and acetonitrile were purchased from Merck (Darmstadt, Germany).

**2. Methods**

**2.1 Preparation of Mixed Standard Solution**

Prepare the following five mixed standard solutions。

Solution 1 was prepared by dissolving accurately weighed substances of cryptochlorogenic acid, isochlorogenic acid A and 20(R)-ginsenoside Rg2 in methanol and diluting to 10ml. The final concentrations in solution 1 were 1μg·ml-1 for cryptochlorogenic acid, 1μg·ml-1 for isochlorogenic acid A, and 5μg·ml-1 for 20(R)-ginsenoside Rg2；

Solution 2 was prepared by dissolving accurately weighed substances of neochlorogenic acid, isochlorogenic acid C, gallic acid, ginsenoside Rc, ginsenoside Rg3 in methanol and diluting to 10ml. The final concentrations in solution 2 were 1μg·ml-1 for neochlorogenic acid, 1μg·ml-1 for isochlorogenic acid C, 1μg·ml-1 for gallic acid, 5μg·ml-1 for ginsenoside Rc, and 5μg·ml-1 for ginsenoside Rg3;

Solution 3 was prepared by dissolving accurately weighed substances of chlorogenic acid, isochlorogenic acid B, ginsenoside Rb2, ginsenoside Rf, ginsenoside Rg2, ginsenoside Rh1, 20(R)-notoginsenoside R2 in methanol and diluting to 10ml. The final concentrations in solution 3 were 1μg·ml-1 for chlorogenic acid, 1μg·ml-1 for isochlorogenic acid B, 5μg·ml-1 for ginsenoside Rb2, 5μg·ml-1 for ginsenoside Rf, 5μg·ml-1 for ginsenoside Rg2, 5μg·ml-1 for ginsenoside Rh1 and 5μg·ml-1 for 20(R)-notoginsenoside R2;

Solution 4 was prepared by dissolving accurately weighed substances of Chrysophanol 1-glucoside, physcion-8-O-glucoside, 20(R)ginsenoside Rg3, 20(R)-ginsenoside Rh1, 20(S)-notoginsenoside R2 in methanol and diluting to 10ml. The final concentrations in solution 4 were 0.2μg·ml-1 for Chrysophanol 1-glucoside, 0.2μg·ml-1 for physcion-8-O-glucoside, 5μg·ml-1 for 20(R)ginsenoside Rg3, 5μg·ml-1 for 20(R)-ginsenoside Rh1and 5μg·ml-1 for 20(S)-notoginsenoside R2;

Solution 5 was prepared by dissolving accurately weighed substances of Ferulic acid, notoginsenoside R1, ginsenoside Re, ginsenoside Rg1, ginsenoside Rb1, ginsenoside Rd, emodin-8-O-β-D-glucoside, aloe-emodin, rhein, emodin in methanol and diluting to 10ml. The final concentrations in solution 5 were 2.04μg·ml-1 for Ferulic acid, 10.18μg·ml-1 for notoginsenoside R1, 9.94μg·ml-1 for ginsenoside Re, 10.21μg·ml-1 for ginsenoside Rg1, 10.06μg·ml-1 for ginsenoside Rb1, 10.14μg·ml-1 for ginsenoside Rd, 0.41μg·ml-1 for emodin-8-O-β-D-glucoside, 0.40μg·ml-1 for aloe-emodin, 0.40μg·ml-1 for rhein and 0.40μg·ml-1 for emodin.

**2.2 Preparation of sample solutions**

Each batch of the FYTF-919 sample was accurately measured and diluted with 50% methanol to 50-fold. Then these solutions were centrifuged (13,000 r/min, 4 ° C) for 10 minutes, and the supernatant was collected for analysis.

2.3 **UHPLC-Q Exactive-Orbitrap MS conditions**

Quality control of FYTF-919, including qualitative and quantitative analysis, were applied by ultrahigh performance liquid chromatography coupled with a Q Exactive hybrid quadrupole-Orbitrap mass spectrometry (UHPLC-Q Exactive-Orbitrap MS) system.

UHPLC analysis was conducted using a Thermo UltiMate 3000 UHPLC instrument (Waltham, MA, USA). The conditions were as follows: Column: ACQUITY UPLC® HSS T3 C_18_ , 2.1 mm×100 mm, 1.8 μm (Waters, USA ); Mobile phase: Acetonitrile (A), water with 0.1% formic acid (B); Column temperature: 35℃, Autosampler temperature: 4℃; Flow rate: 300 μL/min; Gradient: 0-6 min,8%A;6-8min,8-32%A;8-21 min,32%A;21-22min,32-45%A;22-28min,45%A;28-30 min,45-80%A;30-36 min,80%A; Injection volume: 5 μl.

Mass spectrum acquisition was performed using a Q-Exactive hybrid quadrupole-orbitrap mass spectrometer (Thermo Scientific, San Jose, USA) with heat electrospray ionization (HESI) in the negative mode. The main optimized parameters for MS were set as follows: spray voltage, -2.5 kV; capillary temperature, 350 °C; aux gas heater temperature, 350 °C; aux gas flow rate, 15; sheath gas flow rate, 40; sweep gas flow rate, 0; Detection method is Full Mass/dd-MS2; Full Mass resolution 70,000; Scanning range: m/z100-1200; dd-MS2 resolution ratio: 17500.

**2.4** **Method validation for Quantitative experiment**

The mixed standard solution 5 was stepwise diluted with 50% methanol and obtained a series of mixed standard solutions with varying concentrations.

Six concentration levels of them were selected and 10 μl solution were accurately pipetted and analyzed by UHPLC-Q Exactive-Orbitrap MS system. A calibration curve was established by using the concentration (X) as the abscissa, and the peak area (Y) as the ordinate, with the method of least squares applied for linear regression.

The same mixed standard solution was injected into the UHPLC-Q Exactive-Orbitrap MS system six times on the same day for analysis, and the relative standard deviation (RSD) values of the peak areas for each component were calculated separately to evaluate the intra-day precision of the method. The measurements were repeated for 3 consecutive days to assess the inter-day precision of the method. The relative standard deviation (%) was calculated as (standard deviation/mean) × 100.

**3. Results**

**3.1 Similarities of different batches samples**

Chromatogram fingerprint of each batch was shown in Figure 1. The sample similarity analysis was conducted using the "Similarity Evaluation System of Traditional Chinese Medicine Chromatographic Fingerprint" software. As shown in Table 1, the sample similarity of different samples was greater than 0.95, suggesting that the components of FYTF-919 in different batches were almost the same.

**3.2 Calibration Curves and Precision**

Linear relationships of various constituents were shown in Table 2. The results suggested that nine calibration curves represented excellent linearity with high squared correlation coefficient values (R2 ≥ 0.9991) within the detected range.

Both intra-day and inter-day precision were evaluated by the relative standard deviations (RSD). As shown in Table 3, the RSD values of nine components in FYTF-919 were less than or equal to 2.19% (intra-day) and 7.21% (inter-day) respectively, which showed that the precision of the instrument met the experimental requirements.

3.4 Components detected in FYTF-919

Qualitative and quantitative analysis of FYTF-919 were performed by UHPLC-Q Exactive -Orbitrap MS based on the comparison with reference standard solutions. Components in FYTF-919 were presented in Table 4. The quantitative results of representative components in FYTF-919 were presented in Table 5.

**Figure 1. The Chromatogram fingerprints of FYTF-919 with different batches**

**Table 1. Similarities of different batches samples.**

| Batch number | Similarity |
| --- | --- |
| 202109.6 | 0.993 |
| 20211210 | 0.995 |
| 20220419 | 0.992 |

**Table 2. Linear relationships of various components in FYTF-919.**

| Component | Regression equation | Correalation coefficient | Linear range /ng·mL^-1^ |
| --- | --- | --- | --- |
| Notoginsenoside R1 | y = 42.21x - 5.45 | 0.9998 | 39.06~625 |
| Ginsenoside Re | y = 41.11x - 19.02 | 0.9997 | 78.13~1250 |
| Ginsenoside Rg1 | y = 26.19x - 15.29 | 0.9999 | 156.25~2500 |
| Ginsenoside Rb1 | y = 139.62x - 19.30 | 0.9999 | 156.25~2500 |
| Ferulic acid | y = 44.22x - 5.51 | 0.9994 | 125~2000 |
| Rhein | y = 12.11x + 3.01 | 0.9998 | 15.63~250 |
| Emodin-8-O-glucoside | y = 4.41x - 1.70 | 0.9998 | 7.81~125 |

**Table 3. Intra-day and inter-day precision of** **various investigated compounds**

| Compounds | Intra-day precision | | Inter-day precision | |
| --- | --- | --- | --- | --- |
|  | C (ng·mL^-1^, Mean±SD) | RSD/% | C(ng·mL^-1^，Mean±SD) | RSD/% |
| Notoginsenoside R1 | 2096.91±33.67 | 1.61 | 2089.12±27.68 | 1.32 |
| Ginsenoside Re | 1206.55±23.83 | 1.97 | 1160.62±52.75 | 4.55 |
| Ginsenoside Rg1 | 7987.79±88.88 | 1.11 | 7677.68±283.34 | 3.69 |
| Ginsenoside Rb1 | 8229.90±92.75 | 1.13 | 7930.93±263.84 | 3.33 |
| Ferulic acid | 115.44±2.53 | 2.19 | 114.46±1.75 | 1.53 |
| Rhein | 31.31±0.51 | 1.64 | 30.14±1.72 | 5.71 |
| Emodin-8-O-glucoside | 28.59±0.27 | 0.95 | 31.18±2.25 | 7.21 |

**Table 4** **UHPLC-Q Exactive-Orbitrap MS data of 30 components detected in FYTF-919**

| No. | $t_{R}$ /min | [M-H]-/[M+FA-H] | Formula | Identification |
| --- | --- | --- | --- | --- |
|  |  |  |  |  |
| 1 | 1.78 | 169.0132 | C7H6O5 | Gallic acid * |
| 2 | 3.73 | 353.0878 | C16H18O9 | Neochlorogenic acid * |
| 3 | 7.45 | 353.0881 | C16H18O9 | Chlorogenic acid * |
| 4 | 8.72 | 167.0339 | C16H18O9 | Cryptochlorogenic acid * |
| 5 | 8.93 | 179.0340 | C9H8O4 | Caffeic acid |
| 6 | 10.29 | 515.1192 | C25H24O12 | Isochlorogenic acid B |
| 7 | 10.30 | 193.0498 | C10H10O4 | Ferulic acid * |
| 8 | 10.38 | 515.1193 | C25H24O12 | Isochlorogenic acid A |
| 9 | 10.45 | 977.5324 | C47H80O18 | Notoginsenoside R1* |
| 10 | 10.54 | 515.1190 | C25H24O12 | Isochlorogenic acid C |
| 11 | 10.64 | 991.5484 | C48H82O18 | Ginsenoside Re * |
| 12 | 10.70 | 845.4899 | C42H72O14 | Ginsenoside Rg1* |
| 13 | 12.39 | 431.0989 | C21H20O10 | Emodin-8-O-β-D-glucoside * |
| 14 | 14.08 | 845.4905 | C42H72O14 | Ginsenoside Rf |
| 15 | 14.30 | 845.4904 | C42H72O14 | Pseudoginsenoside F11 |
| 16 | 14.74 | 445.1143 | C22H22O10 | Physcion-8-O-β-D-glucoside * |
| 17 | 16.66 | 829.4959 | C42H72O13 | Ginsenoside F2 |
| 18 | 17.29 | 1107.5945 | C42H72O13 | Ginsenoside Rg2 |
| 19 | 17.34 | 1107.5939 | C54H92O23 | Ginsenoside Rb1* |
| 20 | 19.50 | 955.4908 | C48H76O19 | Ginsenoside Ro |
| 21 | 19.72 | 1077.5841 | C53H90O22 | Ginsenoside Rc |
| 22 | 21.44 | 269.0461 | C15H10O5 | Aloe-emodin * |
| 23 | 22.51 | 1077.5845 | C53H90O22 | Ginsenoside Rb2 |
| 24 | 22.72 | 1077.5837 | C53H90O22 | Ginsenoside Rb3 |
| 25 | 24.05 | 283.0259 | C15H8O6 | Rhein * |
| 26 | 24.26 | 945.5422 | C48H82O18 | Ginsenoside Rd* |
| 27 | 30.50 | 269.0459 | C15H10O5 | Emodin * |
| 28 | 30.42 | 829.4959 | C42H72O13 | (S)-Ginsenoside Rg3 |
| 29 | 30.67 | 829.4959 | C42H72O13 | (R)-Ginsenoside Rg3 |
| 30 | 32.27 | 253.0502 | C15H10O4 | Chrysophanol * |

*: Compared with reference substances

**Table 5.** **Concentration of representative components in FYTF-919 (μg/mL, n=3).**

| Components | Concentration |
| --- | --- |
| Notoginsenoside R1 | 2459.68 ± 61.83 |
| Ginsenoside Re | 1380.48 ± 19.48 |
| Ginsenoside Rg1 | 9269.25 ± 250.37 |
| Ginsenoside Rb1 | 9300.57 ± 91.54 |
| Ferulic acid | 86.72 ± 0.56 |
| Rhein | 33.95 ± 0.18 |
| Emodin-8-O-glucoside | 33.43±0.24 |
