# Supplementary material for: Assessing Organoleptic Properties Simulation of a Placebo of Raw Food Material to a Chinese Medicine Oral Liquid FYTF‐919
Source: Biomed Res Int. 2026 Aug 30;2026:9000068. doi: 10.1155/bmri/9000068 (PMC13527160; doi:10.1155/bmri/9000068)
Supplement: Supplementary file 2 [file BMRI-2026-9000068-s002.docx]

Table S1. Sensors and the corresponding volatile components of E-nose

| **Gas sensor array** | **Sensitive volatile components of response** |
| --- | --- |
| S1 | Ammonia and amines |
| S2 | Hydrogen sulfide and sulfides |
| S3 | Hydrogen |
| S4 | Ethanol and organic solvents |
| S5 | Ketones, alcohols, aldehydes, and aromatic compounds |
| S6 | Methane, biogas and natural gas |
| S7 | Flammable gases |
| S8 | Volatile organic compounds |
| S9 | Natural gas |
| S10 | Liquefied gas and flammable gases |
| S11 | Alkanes, ethanol, and smoke |
| S12 | Ethanol and organic solvents |
| S13 | Cooking smoke |
| S14 | Methane and natural gas |

Table S2. Sensors and corresponding taste profiles of E-tongue

| **Taste sensor assay** | **Sensitive taste profile of response** |
| --- | --- |
| S1 | Platinum |
| S2 | Aurum |
| S3 | Palladium |
| S4 | Tungsten |
| S5 | Titanium |
| S6 | Argentum |

**Table S3** Univariate regression analysis of factors influencing placebo odor score

| Variables | B | 95% CI | *P* value |
| --- | --- | --- | --- |
| Age | -0.006 | (-0.077, 0.064) | 0.851 |
| Male | 1.244 | (-1.867, 4.356) | 0.416 |
| Medical worker | -1.167 | (-4.183, 1.849) | 0.431 |
| History use of TCM | -0.337 | (-4.101, 3.427) | 0.854 |

**Table S4** Univariate regression analysis of factors influencing placebo taste score.

| Variables | B | 95% CI | *P* value |
| --- | --- | --- | --- |
| Age | 0.002 | (-0.064, 0.067) | 0.960 |
| Male | 0.156 | (-2.771, 3.082) | 0.913 |
| Medical worker | -1.583 | (-4.330, 1.164) | 0.245 |
| History use of TCM | 0.158 | (-3.331, 3.647) | 0.926 |

**Table S5** Univariate regression analysis of factors influencing FYTF-919 taste score.

| Variables | B | 95% CI | *P* value |
| --- | --- | --- | --- |
| Age | -0.021 | (-0.132 0.089) | 0.689 |
| Male | 0.909 | (-2.209 4.027) | 0.551 |
| Medical worker | 1.879 | (-1.149 4.906) | 0.211 |
| History use of TCM | 1.446 | (-1.904 4.797) | 0.380 |
